# Quantification of Pharmacokinetic Profiles of PD-1/PD-L1 Antibodies by Validated ELISAs

**DOI:** 10.3390/pharmaceutics12060595

**Published:** 2020-06-26

**Authors:** Sara Zalba, Ana M. Contreras-Sandoval, Eva Martisova, Reno Debets, Christian Smerdou, María Jesús Garrido

**Affiliations:** 1 Department of Pharmacy and Pharmaceutical Technology, School of Pharmacy, University of Navarra, 31008 Pamplona, Spain; szalbaot@unav.es (S.Z.); acontreras.1@alumni.unav.es (A.M.C.-S.); 2 Department of Molecular Oncology, Moffitt Cancer Center & Research Institute, Tampa, FL 33612, USA; 3 Division of Gene Therapy and Regulation of Gene Expression, CIMA Universidad de Navarra and Instituto de Investigación Sanitaria de Navarra (IdISNA), 31008 Pamplona, Spain; emartisova@alumni.unav.es (E.M.); csmerdou@unav.es (C.S.); 4 Laboratory of Experimental Tumor, Medical Oncology Department, Erasmus MC Cancer Institute, 3015 GD Rotterdam, The Netherlands; j.debets@erasmusmc.nl

**Keywords:** PD-1, PD-L1, ELISA, validation, pharmacokinetics

## Abstract

Immunotherapy has changed the paradigm of cancer treatments. In this way, several combinatorial strategies based on monoclonal antibodies (mAb) such as anti (a)-PD-1 or anti (a)-PD-L1 are often reported to yield promising clinical benefits. However, the pharmacokinetic (PK) behavior of these mAbs is a critical issue that requires selective analytical techniques. Indeed, few publications report data on a-PD1/a-PD-L1 exposure and its relationship with therapeutic or toxic effects. In this regard, preclinical assays allow the time profiles of antibody plasma concentrations to be characterized rapidly and easily, which may help to increase PK knowledge. In this study, we have developed and validated two in-house ELISAs to quantify a-PD-1 and a-PD-L1 in plasma collected from tumor-bearing mice. The linear range for the a-PD-1 assay was 2.5–125 ng/mL and 0.11–3.125 ng/mL for the a-PD-L1 assay, whereas the intra-and inter-day precision was lower than 20% for both analytes. The PK characterization revealed a significant decrease in drug exposure after administration of multiple doses. Plasma half-life for a-PD-1 was slightly shorter (22.3 h) than for a-PD-L1 (46.7 h). To our knowledge, this is the first reported preclinical ELISA for these immune checkpoint inhibitors, which is sufficiently robust to be used in different preclinical models. These methods can help to understand the PK behavior of these antibodies under different scenarios and the relationship with response, thus guiding the choice of optimal doses in clinical settings.

## 1. Introduction

Therapeutic monoclonal antibodies (mAb) represent one of the most promising strategies to treat different types of diseases, including cancer [1]. In this context, mAbs against certain upregulated molecules in cancer cells and the tumor microenvironment have opened up new mechanisms to achieve tumor regression [2]. Cancer immunotherapy based on the modulation of the immune system using certain mAbs has substantially contributed to achieving relevant clinical outcomes [3].

In this regard, immune checkpoints (ICs) such as Programmed Death-1 receptor (PD-1) and its endogenous ligands, PD-L1 (B7-H1) and PD-L2 (B7-DC), have been shown to be validated targets for cancer treatments [4]. PD-1, known as CD279, is a type I transmembrane protein belonging to the CD28 family of immune regulatory receptors, mainly expressed in activated T-cells, which promotes their proliferation and self-tolerance [5]. Indeed, expression of this receptor declines as the antigen is successfully eliminated. On the other hand, PD-L1 (B7-H1), a transmembrane glycoprotein included in the B7 family of immune regulatory molecules [6], is constitutively expressed on inflammatory-activated immune cells such as macrophages, T-and B cells, and endothelial and intestinal epithelial cells and is also inducible in many other cells, particularly cancer cells in the presence of certain pro-inflammatory stimuli [7].

**Figure 1:**
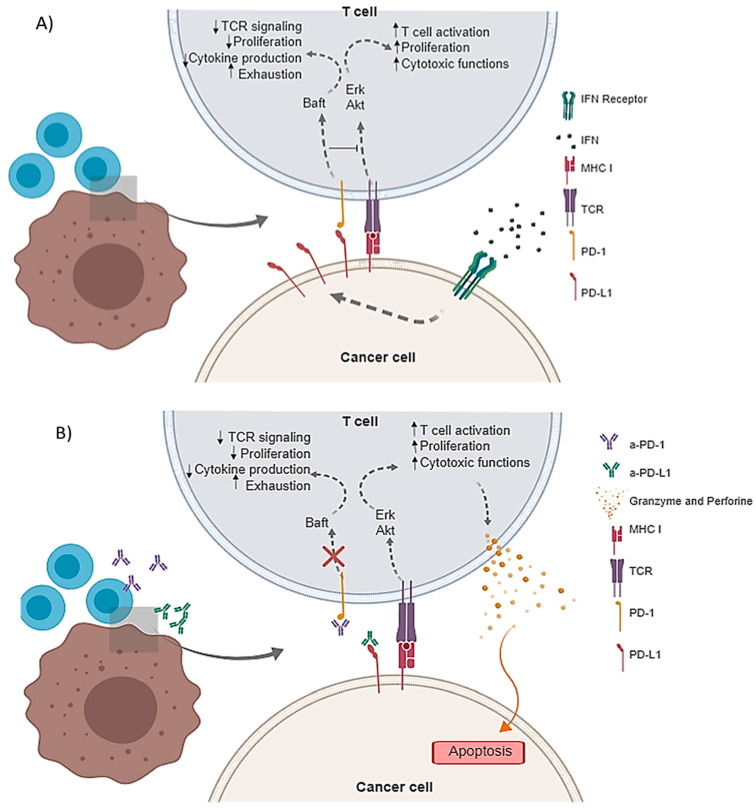
Schematic representation of PD-1/PD-L1 axis. (**A**) PD-L1 overexpression in response to Interferon Ɣ (IFNƔ) leads to the T cell exhaustion and anergy; (**B**) with the use of a-PD-1 or a-PD-L1 antibodies, T cells are able to exert their cytotoxic effector activity and promote cancer cell apoptosis.

PD-1/PD-L1 binding promotes the reduction of T-cell proliferation, inducing immune suppressor cytokine production that leads to lymphocytes apoptosis, anergy, and functional exhaustion, as is depicted in Figure 1 [5,8]. This mechanism involved in avoiding auto-immunity is responsible for tumor immune escape. In that sense, the blockade of this PD-1/PD-L1 interaction by specific mAbs (a-PD-1 or a-PD-L1) has demonstrated tumor eradication and has contributed to the enhancement of other cancer therapies [9,10].

Currently, there are several PD-1/PD-L1 inhibitors approved for clinical use: Pembrolizumab, Nivolumab, and Cemiplimab to block PD-1, and Atezolizumab, Durvalumab, and Avelumab for PD-L1 [9,11].

However, despite their therapeutic effects, inter-individual variability is very high, ranging from responders to non-responders [12]. This characteristic makes difficult the establishment of an adequate relationship between antibody exposure and efficacy, thereby limiting the administration of adequate dosing regimens. Thus, characterization of the pharmacokinetic (PK) behavior of these mAbs would be very useful in guiding individual doses to achieve tumor regression in each patient. At this point, the paucity of studies focusing on the PK characterization of IC inhibitors (ICIs) highlights the need to know more about their behavior in order to help in predicting clinical responses.

Preclinical models have contributed to studying the PK of different mAbs and the impact on response. Indeed, physiological PK models seem to improve the characterization of drug behavior, providing the most relevant platform to achieve appropriate human dose escalation [13]. However, the studies on the biodistribution of these biological agents in animals have been done in many cases by imaging experiments [14,15,16], highlighting the need to develop selective and sensitive analytical techniques to quantify plasma and tissue antibody concentrations accurately. In this way, the enzyme-linked immunosorbent assay (ELISA) is the most popular analytical assay to measure mAbs in different biological matrices [17]. To our knowledge, no publications reporting ELISA validation for these ICIs in preclinical tumor mice models are currently available.

Therefore, this work aims to develop and validate two easy-to-use ELISAs to quantify a-PD-1 and a-PD-L1 plasma concentrations collected from tumor-bearing mice and determine the PK profiles and main parameters of these agents.

## 2. Materials and Methods

### 2.1. Materials

Recombinant mouse PD-1-Fc Chimera, PD-L1/B7-H1-Fc Chimera Protein, and a-Goat IgG HRP-conjugated antibody were purchased from R&D Systems^®^ (Minneapolis, MN, USA). 3,3’,5,5’-tetramethylbenzidine (TMB) was obtained from Millipore^®^ (Burlington, MA, USA). Bovine Serum Albumin (BSA), sulfuric acid, streptavidin-peroxidase, and Tween-20 were obtained from Sigma Aldrich^®^ (St. Louis, MO, USA). DPBS without calcium and magnesium and fetal bovine serum (FBS) were purchased from Gibco^®^ (Madrid, Spain). Rat a-mouse-PD-1 mAb (CD279; clone RMP1-14) and rat a-mouse PD-L1 (B7-H1, clone 10F.9G2) were obtained from BioXCell^®^ (West Lebanon, NH, USA). The Goat a-Rat IgG, (H+L) antibody, Biotin Conjugated was purchased from Thermo scientific^®^ (Waltham, MA, USA).

### 2.2. Methods

#### 2.2.1. ELISA Development and Validation

##### Matrix Selection

In order to quantify plasma levels of a-PD-1 and a-PD-L1, two different sandwich ELISAs were developed and validated in our laboratory. To that end, a plasma matrix obtained from C57/B6J drug-free mice was used to prepare standard samples. Briefly, blood samples were collected in heparinized-EDTA tubes and centrifuged at 4°C for 3500 rpm for 15 min to obtain the plasma, which was kept at −20°C until use.

The stock solution for ELISAs was prepared using this drug-free plasma diluted 1:150 (*v/v.*) in incubation buffer (IB; 0.05% Tween-20, 0.1% BSA in DPBS) obtaining the matrix buffer (MB). This MB was spiked with different known drug concentrations to prepare the standard samples and the analytical control, which corresponded to free-drug MB.

##### Calibration Standard Curves

Two working solutions or stocks, 500 ng/mL for a-PD-1 and 250 ng/mL for a-PD-L1, were freshly prepared in MB to build the corresponding calibration standard curves.

##### a-PD-1 ELISA

**Figure 2:**
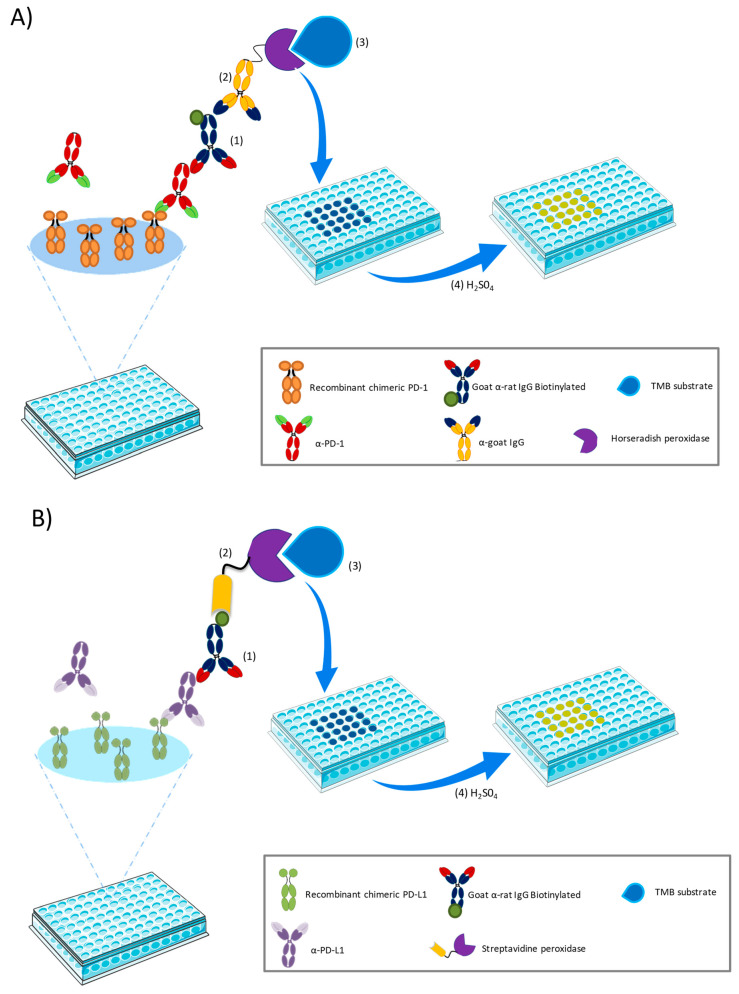
Schematic representation of the sandwich ELISA developed and validated in the present work. (**A**) ELISA to quantify a-PD-1 in plasma; numbers correspond to the consecutive steps: (1) addition of secondary goat α-rat IgG, (2) incubation with α-goat IgG HRP-conjugated antibody, (3) incorporation of TMB substrate, (4) stopping of the reaction with H_2_SO_4_. (**B**) ELISA to detect a-PD-L1 in plasma;numbers correspond to the different steps of the ELISA: (1) addition of secondary goat α-rat IgG biotin conjugate, (2) incubation with streptavidin-peroxidase, (3) incorporation of TMB substrate, (4) stopping of the reaction with H_2_SO_4_.

Figure 2A shows the schematic representation of the developed a-PD-1 ELISA sandwich method. Briefly, 24 h before performing the assay, a flat-bottomed 96-well Maxisorp microplate (NUNC, Thermoscientific, Rochester, NY, USA) was coated with 2 μg/mL of recombinant mouse-PD-1 capture fusion protein diluted in DPBS and incubated at 4°C overnight. Afterward, the plate, washed twice with 200 μL/well of DPBS, was blocked with 200 μL/well of IB for 1 h at room temperature (RT) to prevent unspecific reactions. Then, the plate was washed five times using 200 µL/well of washing buffer (0.05% Tween-20, DPBS; WB), and the standards and experimental samples (100 μL/well) were added and incubated for 2 h at RT. For the labeling, the plate was washed and treated for 1 h with a secondary goat a-rat IgG antibody (1:20.000, *v/v.* IB). In order to amplify this signal, a-Goat IgG HRP-conjugated antibody was added (1:5.000, *v/v.* IB) and incubated for 1 h at RT.

The plate was washed and revealed with 100 μL/well of TMB for 3 min. This reaction was stopped by adding 50 μL/well of sulphuric acid (2 N), and the absorbance was read at 450 nm in PowerWave™ XS Microplate Reader from BioTek Instruments, Inc (Winooski, VT, USA).

##### a-PD-L1 ELISA

The protocol for this ELISA (Figure 2B) was similar to the a-PD-1 ELISA described above. Aliquots of 2 μg/mL of recombinant mouse-PD-L1 capture fusion protein diluted in DPBS (100 µL/well) were placed in a flat-bottomed 96-well Maxisorp microplate and incubated in darkness overnight at 4°C. After being washed twice with DPBS, wells were blocked with 200 μL/well of IB for 1 h at RT. Then, samples and standards prepared with MB (100 μL/well) were added and incubated for 2 h at RT. Immediately after, the plate was washed and incubated with a Goat a-rat IgG antibody (1:100,000, *v/v.* in IB) for 1 h at RT, followed by another 1 h incubation with Streptavidin-peroxidase (1:50,000 *v/v.* in IB) at RT in darkness. The plate was revealed with 100 μL/well of TMB for 3 min. This reaction was stopped by adding 50 μL/well of sulphuric acid (2 N), and the absorbance was read at 450 nm.

#### 2.2.2. ELISA Validation

The criteria for the analytical assay validation were performed in accordance with the current recommendations for bioanalytical methods: linearity, accuracy, precision, and reproducibility [18,19].

##### Optimization of the Labeling Signal

Several dilutions of the secondary antibody were evaluated to optimize the signal. In the case of a-PD-1, the a-Goat IgG HRP-conjugated antibody was tested at three dilutions (1:2500, 1:5000, and 1:10,000), whereas for a-PD-L1, the Goat a-rat IgG antibody was assayed at two dilution levels (1:75,000 and 1:100,000).

##### Linear Range

Several standard samples were prepared to establish and analyze the linear range of calibration curves. The stock solutions, 500 ng/mL for a-PD-1 and 250 ng/mL for a-PD-L1, were used to prepare serials of drug concentrations in MB for the standard curves. The parameters, slope, and intercept corresponding to the linear regression curve (y= ax+b; where y, represents the signal and x, the independent variable or antibody concentration) were calculated for each assay and statistically analyzed according to the intra- and inter-day variability [19,20]. The correlation was established by r^2^≥0.98.

##### Determination of the Limits of Quantification and Detection

The sensitivity of the assay is often described by the Limit Of Quantification (LOQ), which is the lowest point at which the analyte or therapeutic molecule can be accurately measured. The Limit of Detection (LOD) was also determined, being considered as the lowest quantity of a substance that can be distinguished from the absence of that substance (a blank value). To establish the corresponding values for LOQ and LOD, ten replicates of controls or MB samples were analyzed. The following formulas were applied to calculate both values:

LOQ = (mean/SD) × 10(1)

LOD = (mean/SD) × 3.3(2)

where mean corresponds to the average value of control MB samples and SD to their standard deviation [21].

##### Precision and Accuracy

The precision parameter accounts for the repeatability (intra-day or within-day variability) and reproducibility (inter-day or between-day variability). To determine the repeatability, three specific concentration levels covering the linear range were selected for both assays: 62.5, 31.3, and 3.9 ng/mL for a-PD-1 and 3.125, 0.781, and 0.195 ng/mL for a-PD-L1.

Each sample, prepared in triplicate, was analyzed on the same day and on three different days. The first analysis determined the assay precision referred to the intra-day variability and the following analyses were for the inter-day variability. In both cases, the value was calculated by the following formula:

Precision (%CV) = (SD/Mean) × 100(3)

Accuracy, expressing the closeness between the real concentration and the measured value according to the developed assay, was performed and calculated with this formula:

Accuracy (% Mean bias) = (Observed concentration/Theoretical concentration) × 100(4)

Therefore, the imprecision of the determination was:

Imprecision = Accuracy − 100(5)

According to the guidelines, precision should be < 20%, and accuracy should be within ± 20% [18,19].

##### Stability and Plate Drift

The stability ofsamples was determined using three concentration levels of a-PD-1,(62.5, 31.3, and 3.9 ng/mL) and of a-PD-L1 (3.125, 0.781, and 0.195 ng/mL).These samples were analyzed just after preparation, kept at 4 °C and analyzed again one month later, to evaluate the impact of storage.

In addition, the plate drift variability was also analyzed to establish whether the time elapsed during plate-well preparation (samples plus reagents) couldaffect the results.To that end, some samples were added in the first-row wells, and the same samples were placed in the last row wells after completing the plate. The absorbance was statistically compared to determine possible differences.

#### 2.2.3. a-PD-1 and a-PD-L1 Pharmacokinetics

In vivo experiments were performed according to the European animal care regulations andprotocols approved by the Ethics Committee of the University of Navarra (Reference number 023/17, aprobbed on 6 April 2017 and reference number protocol 129/16, aprobbed on 15 November 2016). Female C57BL/6 mice (5-week old, weighing approximately 20 g supplied by Harlan, Barcelona, Spain) were housed in plastic cages under standard and sterile conditions, 25 °C, 50% relative humidity, 12 h dark/light, with water and food *ad libitum*. Two tumor cell lines, TC-1/A9 and B16-OVA, were used for this study. ICIs’ plasma levels were quantified with the previously validated ELISAs and analyzed by a linear regression approach, using log-transformed data and considering a biphasic disposition. The time profiles of the plasma concentrations for the two antibodies were built using the average value corresponding to the three mice used at each time point (see Section 2.2.3.1 and Section 2.2.3.2). The area under the plasma concentration versus time curve (AUC_0−∞_) was calculated by the linear trapezoidal method. For the extrapolated area, AUC_72h−∞_, the last concentration C_72h_ was divided by the slope (*k*) obtained from the terminal portion of the curve that determines the rate of drug decline:

(6) $$\;{\left[AUC\right]}_{72h}^{\infty }=C72h/k$$

other parameters such as half-life, clearance (*Cl*), and volume of distribution (*V_d_*) were estimated according to the equations *t*_1/2_ =0.693/*k*, *CL* = Dose/AUC_0−∞_ and *Vd = Cl /k*, respectively.

The plots were built in RStudio (version3.4.3) using the ggplot2 package.

##### 2.2.3.1. Characterization of a-PD-1 Pharmacokinetics

A total of nine C57/B6J female mice were inoculatedwith TC-1/A9, a cervix carcinoma murine cell line, kindly provided by Dr. Pedro Berraondo (CIMA, Pamplona, Spain). Cells, maintained in standard culture conditions (5% CO_2_ and 37 °C), were grown in RPMI 1640 with GlutaMAX supplemented with FBS (10%(*v/v*)), Penicillin/Streptomycin(1%(*v/v*)), Geneticin (0.4 mg/mL), and 2-mercaptoethanol (0.05 mM). Mycoplasma was regularly tested for with a luminescence assay (LONZA, Basel, Switzerland).

**Figure 3:**
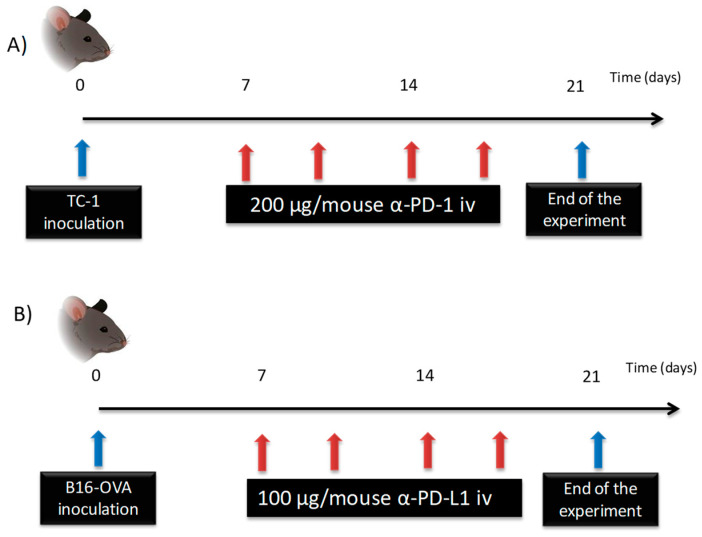
Schematic representation of the dose regimen used for immune checkpoint inhibitors(ICIs): (**A**) a-PD-1 was intravenously (iv) administered to TC-1 tumor-bearing mice; (**B**) a-PD-L1 was iv injected to B16-OVA tumor-bearing mice.

Tumor cells (1 × 10^5^ cells/100 µL PBS) were subcutaneously inoculated into the right flank of animals. One week later, when the average tumor diameter was approximately 5 mm, mice were treated (*n* = 9), receiving, intravenously, 100 µL of drug solution via tail vein and four administrations of 200 µg/mouse a-PD-1 dose every 72 h, as shown in Figure 3A. 100 µL blood samples were collected into heparin tubes by facial puncture at 10, 30 min and 1, 3, 8, 24, and 72 h after the first and fourth dose administration. To that end, animals were randomly divided into three subgroups (*n* = 3) to obtain a maximum of three blood samples per animal during each cycle of treatment. All samples were centrifuged at 3500 rpm for 15 min at 4 °C, and plasma was kept at −20 °C until analysis. These samples were diluted 1:150, as the calibration curves, followed by another 1:2 dilution, in the case of the three first time points.

##### 2.2.3.2. Characterization of a-PD-L1 in an In Vivo Model

The B16-OVA melanoma murine cell line, kindly provided by Dr. Sandra Hervás-Stubbs (CIMA, Pamplona, Spain), was used to establish the in vivo tumor model. Cells, maintained in standard culture conditions, were grown in RPMI 1640 with GlutaMAX supplemented with FBS (10%(*v/v*)), Penicillin/Streptamycin(1%(*v/v*)), Geneticin (0.4 mg/mL), 2-mercaptoethanol (0.05 mM), and Hepes (20 mM). Geneticin was added to culture medium during the two first passages to assure OVA expression.

Tumor cells at a density of 5 × 10^5^ cells/100µL PBS were subcutaneously injected into the right flank of the animal. One week later, when tumors reached a diameter of approximately 5 mm, mice (*n* = 9) were intravenously injected with 100 µL of drug solution via the tail vein. The treatment consisted of four administrations of 100 µg/mouse of a-PD-L1 every 72 h (Figure 3B).

Animals, divided into three subgroups (*n* = 3) were randomly assigned to the different time points for a maximum of three blood sample collections in each cycle of treatment per mouse. Blood samples (100 µL) were collected by facial puncture at 10, 30 min and 1, 3, 8, 24, and 72 h after first and fourth dose administration into heparin tubes. Plasma samples were kept at −20 °C until analysis. These plasma samples were diluted 1:150 followed by an extra 1:20 dilution for the first dose and time points.

#### 2.2.4. Statistical Analysis

All data were expressed as mean ± SD and coefficient of variation (CV) using GraphPad Prism 6 (GraphPad Software, San Diego, CA, USA). The Student t-test or U-Mann-Whitney, depending on the number of data per group, were applied for statistical comparison of the two groups. The significance level was set at 0.05.

## 3. Results

### 3.1. ELISA Validation

#### 3.1.1. Optimization of the Secondary Antibody

**Figure 4:**
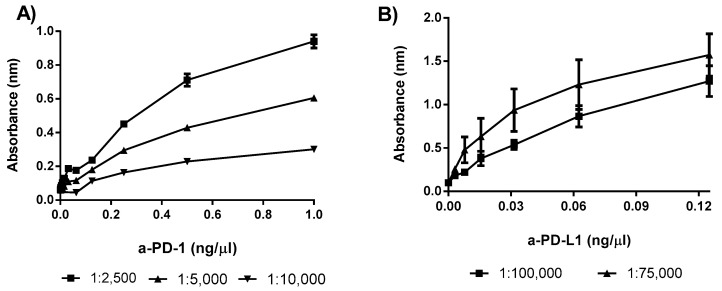
Different antibody dilutions performed to optimize the corresponding ELISA: (**A**) a-PD-1 assay with several a-Goat IgG HRP-conjugated dilutions; (**B**) a-PD-L1 assay with different concentrations of rat a-Goat IgG antibody. Data correspond to 3 independent experiments.

The optimization of the secondary antibody dilution was the first issue to tackle. Several curves, as shown in Figure 4, were built using different antibody dilutions in order to find the most adequate slope for the relationship between ICI concentration and analytical signal. The dilution selected for the a-PD-1 assay was 1:5000 and for a-PD-L1 was 1:100,000, as both provided a linear relationship. For the other conditions 1:2500 (a-PD-L1) and 1:75,000 (a-PD-1), a rapid saturation of the signal was found, thus posing serious difficulties for antibody quantification.

#### 3.1.2. Linearity

**Figure 5:**
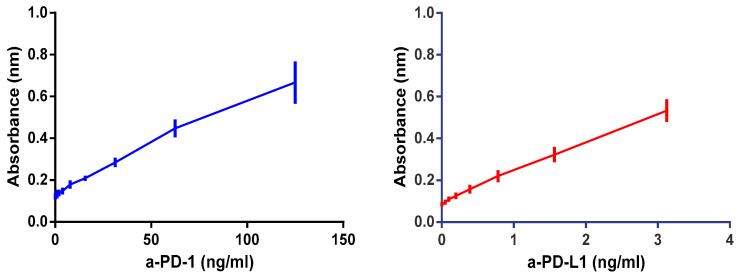
Average and standard deviation of 6 independent standard curves of a-PD-1 in blue and a-PD-L1 in red.

The linear range for a-PD-1 was established between 125 and 2.5 ng/mL. However, for a-PD-L1, this range was narrower, between 3.125 and 0.10 ng/mL .Several standard curves were prepared in these ranges, and the linearity was determined by the correlation parameter *r*^2^, which turned out to be ≥ 0.98, as is represented in Figure 5. In addition, Table 1 lists the individual standard curve equations obtained in the linear range for both antibodies, together with the corresponding correlation value, demonstrating the linear relationship between antibody concentrations and the absorbance signal.

In addition, Table 2 reports the slopes and intercepts for each standard curve prepared for both antibodies, a-PD-1 and a-PD-L1 respectively. The CV calculated for these parameters was in all cases < 20%, suggesting a low variability associated with the ELISA. Non-statistical differences across the parameters estimated for the standard curves corresponding to each ICI were found.

#### 3.1.3. LOQ and LOD

LOD for a-PD-1 and a-PD-L1 were established at 0.12 ng/mL and 0.03 ng/mL respectively, whereas LOQ was determined as 2.5 ng/mL for a-PD-1 and 0.11 ng/mL for a-PD-L1.

#### 3.1.4. Precision and Accuracy

Inter-and intra-day accuracy and imprecision for both ELISAs are reported in Table 3. The selected drug concentrations were in the range of high, medium, and low, according to the linear range. All samples were prepared following the previously described methodology. For precision, the intra- and inter-day bias ranged from 2.91 to 9.68% and 5.92 to 9.15%, respectively, in the case of a-PD-1. In the case of a-PD-L1, the range was between 6.56 and 5.31% for intra-day and between 7.75 and 7.30% for inter-day as is reported below.

The variability or CV associated with the different antibody concentrations was < 10%, although for the lowest a-PD-1 plasma concentration, there was a tendency for it to be higher, but this is not expected to have any particular impact on further results. In addition, the imprecision of the assays was < 20%.

In order to evaluate the impact that the dilution of the highest concentration may have on the results from both assays, the accuracy of serial dilutions prepared with the same sample was determined. Results in Table 4 show that the dispersion was < 10%. This finding, together with imprecision percentages, supports the notion that the ELISA procedures were sufficiently accurate to be applied in in vivoplasma samples.

#### 3.1.5. Stability and Plate Drift

The impact of plate drift was evaluated using the highest plasma concentration of a-PD-1 (62.5 ng/mL) and a-PD-L1 (3.125 ng/mL), respectively. Aliquots of these samples were placed in the first and the last row. Results for a-PD-1 did not differ statistically between the first and the last wells, which were totally comparable (61.94 ± 6.9 ng/mL vs. 58.63 ± 5.49 ng/mL). This finding was similar to a-PD-L1, where the concentration in the first and the last rows, 3.383 ± 0.81 ng/mL vs. 3.469 ± 0.46 ng/mL, were almost exactly the same.

On the other hand, since in vivo plasma samples are often kept on ice during hours before the performance of the analytical assay, the antibody stability in this matrix was evaluated. Several plasma samples using high, medium, and low concentrations of the two antibodies were prepared. Table 5 shows the results for these assays. No statistically significant differences were found in any case, thus supporting adequate stability during at least one month.

### 3.2. InVivo Characterization of Plasma Immune Checkpoint Inhibitor Levels

#### 3.2.1. a-PD-1 Plasma Time Course Profile

**Figure 6:**
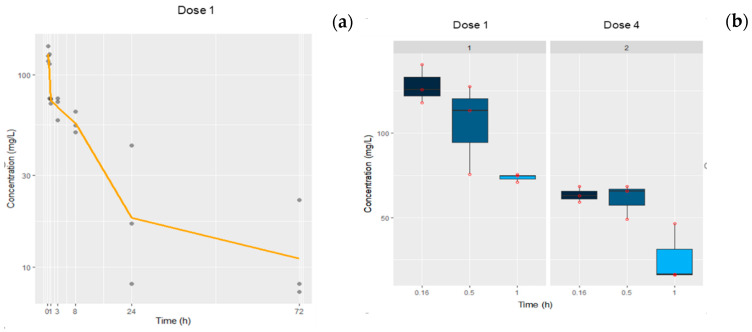
(**a**) Time course profile of a-PD-1 plasma concentrations collected from tumor-bearing mice at different time points after receiving the first dose (*n* = 3/time point). Experimental data are represented by points and the solid line corresponds to the mean (left panel). (**b**) Box plots represent the plasma concentrations at 10, 30, and 60 min after the first and fourth administrations; symbols show the observed data (right panel).

Blood samples were collected at different time points between 10 min and 72 h after dose administration. Figure 6, left panel, shows the time profile of a-PD-1 plasma concentrations after the first iv administration to TC-1 tumor-bearing mice.

The half-life calculated by a non-compartmental approach was approximately 25 h, with a drug exposure expressed as AUC_0−∞_ of 2427.35 µg·h/mL (Table 6). The right panel in Figure 6 shows a comparison between plasma concentrations at 10, 30, and 60 min after the 1^st^ and the 4^th^ dose administration. An important change in the exposure to the drug achieved after repeated dose was observed. Indeed, the mean plasma levels at 10 min after the first dose was double (128.01 ± 11.52 µg/mL) compared to after the fourth dose (63.43 ± 4.59 µg/mL) (*p* < 0.05). Note that for the fourth dose, most of the plasma concentration values were below the limit of quantification.

#### 3.2.2. a-PD-L1 Plasma Time Profile

**Figure 7:**
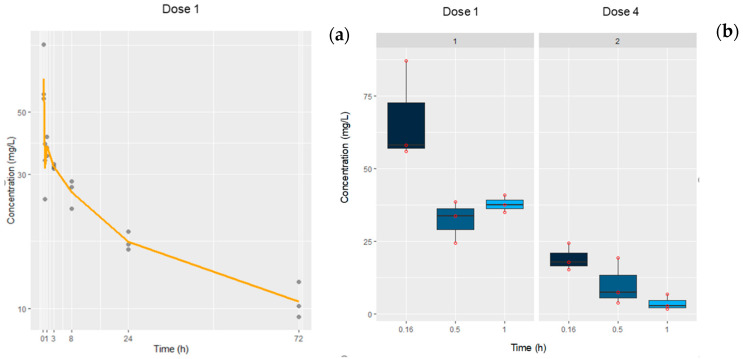
(**a**) Time course profile of a-PD-L1 plasma concentrations collected from tumor-bearing mice at different time points after receiving the first dose (*n* = 3/time). Experimental data are represented by points, and solid lines correspond to the mean (left panel). (**b**) Box plots represent the plasma concentrations at 10, 30, and 60 min after the first and fourth administrations; symbols show the observed data (right panel).

Figure 7 shows the time course profile of a-PD-L1 plasma levels quantified in B16-OVA tumor-bearing mice after receiving 100 µg of mAb/mouse iv. The treatment consisted of four doses administered every 3 days. Plasma levels were quantified after the first and fourth doses to determine possible changes during each cycle. In this case, the half-life calculated also by a linear regression was 46.7 h, slightly longer than for a-PD-1, although more data would be needed to increase the robustness of these results (Table 7). In addition, as occurred with a-PD-1, data after the fourth dose showed a reduction in antibody plasma levels at 10 min (19.13 ± 4.19 mg/L) up to 3.5 times in comparison with the first dose (66.99 ± 17.4 mg/L) (*p* < 0.001), as is observed in the right panel of Figure 7.

Drug exposure in this case was lower than for a-PD-1, because the dose administered to mice was 100 µg instead of the 200 µg used for a-PD-1, and due to the high variability, it was difficult to confirm a correlation between dose and drug-exposure.

## 4. Discussion

Immune checkpoint inhibitors currently represent the most promising therapeutic agents in oncology. However, unfortunately, these mAbs are associated with a high inter-individual variability [12]. To investigate these differences in detail, a particular effort in developing animal models and analytical assaysis required. In the case of therapeutic antibodies, ELISA is one of the most commonly used techniques, due to its specificity and precision [17].

Although the methodology applied for both murine antibodies was similar, they showed a very different linear-range: 125–2.5 ng/mL for a-PD-1 and 3.125–0.11 ng/mL for a-PD-L1. This might be explained by the difference in the antibody isotype (IgG2a and IgG2b, respectively) or their affinity for the recombinant protein used to cover the ELISA plates. Indeed, in the a-PD-1 ELISA, unlike the a-PD-L1, an additional step including two secondary antibodies was necessary to properly amplify the signal. Even with this extra step, the slope for the a-PD-1 assay proved to be 30 times lower than for the a-PD-L1, suggesting a lower sensitivity. Nevertheless, both assays were validated and shown to be sufficiently robust to evaluatein vivo plasma samples.

Thus, we have characterized the time-course profiles of a-PD-L1 and a-PD-1 plasma concentrations after repeated iv administrations to mice. Note that we have used tumor-bearing mice to evaluate the PK behavior of antibodies in contrast to other studies in which mice were tumor-free [22,23]. Furthermore, few papers are available in the literature regarding the PK characterization of a-PD-1 and a-PD-L1. These characterizations are based mainly on imaging methods and even radioactivity levels [14,24]. In the studies on a-PD-1 or a-PD-L1 quantification in different matrices, most of the data correspond to human or primate serum samples with clear differences in the results across ELISA data [19,25,26,27].

Indeed, for a-PD-1 ELISA, there is no clear consensus on LOQ values for human mAbs. For example, Pluim et al. have reported 2 ng/mL, a very similar cipher to the result found here (2.5 ng/mL) [25], while other authors such as Fu et al. [26] or Puszkiel et al. [19] have reported 19.531 ng/mL and 5 µg/mL, respectively. Note that there are some important differences in the procedures: (i) Pluim and co-workers used a-PD-1 to cover the plates, whereas Puszkieland co-workers used a human recombinant PD-1/Fc; (ii) Pluim et al. found important serum interference in the assay, while this was negligible in the assay reported by Puszkiel et al.; and finally, (iii) Pluim and co-workers used luminescence for the quantification in contrast to the UV absorbance used by Puszkiel et al. No detailed information about the methodology used by Fu et al. is reported, making any comparison with the previous authors difficult. In any case, our assay methodology is closer to Puszkiel’s work, but the differences in the a-PD-1 antibody and the recombinant protein might explain the discrepancy in the parameters.

On the other hand, few papers in the literature provide information about a-PD-L1 plasma level quantification by ELISA. In that sense, Deng and co-workers have reported two quantitative analytical techniques to measure two new a-PD-L1 molecules [27]. These authors obtaineda linear range, 0.391–25 ng/mL, larger than the one found in this work, using a dilution factor of 1:20 for mouse serum samples. However, although the lowest concentration was 0.391 ng/mL, the LOQ was established at 7.8 ng/mL, higher than 0.11 ng/mL found here; in contrast, the intra- and inter-assay precision were very similar, even when Deng and co-workers used human proteins, which differ from those used in this work, a recombinant mouse-PD-L1 chimerato capture a-PD-L1 rat antibody.

Therefore, the methodology as well as the proteins chosen to detect and quantify a-PD-1/a-PD-L1 can influence the results. In any case, the ELISAs developed in the present work were in line with the validation parameters and results found in the literature.

Regarding in vivo ELISAs application, the PK behavior of these two antibodies was quite similar. Thus, the volume of distribution for both molecules was similar reflecting the interstitial and vascular volume, as is reported for this type of therapeutic agent. Regarding mAb clearance, Deng et al. have reported 0.0483 mL/h for a 1 mg/kg dose, similar to the value found in this work for a-PD-L1 at 5 mg/kg [27]. In the case of the terminal half-life, both mAbs displayed values shorter than two days but longer than those reported by Deng. Note that these authors studied the PK of a-PD-L1 in BALB/c tumor-free mice, and non-interindividual variability was graphically observed. A greater amount of data would be desirable to better determine mAb PK characterization in blood, as well as in other tissues for biodistribution. However, the validated analytical assays presented here provide valuable information, supporting the variability observed in animals that are in line with that found in patients and, more importantly, to understand the relationship between drug exposure and therapeutic effects. In this way, the evaluation of the fourth dose has demonstrated a reduction in the exposure to these antibodies. There are several explanations for this lower exposure after repeated doses such as an immunogenic effect or a target-mediated drug disposition. Both mechanisms have been reported for many biological agents affecting the PK behavior in a serious manner such as accelerating plasma clearance [28,29]. This finding may support the low efficacy that these antibodies trigger in monotherapy and the high inter-individual variability, which is also found in patients [12]. In fact, our group already tested the antitumoral effect in monotherapy of both ICIs, where the effect was scarce (see supplementary Figure S1A,B).

Thus, the understanding of the relationship between drug exposure and response for a therapeutic agent in preclinical models might help establish optimal dose regimens in patients. These models represent an interesting translational platform to explore dose regimens and effects under different scenarios [13,30].

In conclusion, in this study, we have developed and validated two ELISAs to determine a-PD-1 and a-PD-L1 plasma levels in tumor-bearing mice. These assays have allowed the PK profiles for both ICIs to be characterized after multiple dosing and provide a basis for applying this methodology to explore ICI levels in other matrices.

## Figures and Tables

**Table 1:** Regression curves and *r*^2^ calculated for each calibration curve and for the average curve.

PD-1		PD-L1	
Curve Equation	r^2^	Curve Equation	r^2^
y = 0.005645x +0.1341	0.99	y = 0.1403x + 0.10950	0.99
y = 0.003423x + 0.1359	0.99	y = 0.1386x + 0.09234	0.99
y = 0.003899x + 0.1414	0.99	y = 0.1473x + 0.09648	0.99
y = 0.004170x + 0.1156	0.99	y = 0.1382x + 0.08988	0.99
y = 0.004084x + 0.1192	0.98	y = 0.1476x + 0.11510	0.99
y = 0.004636x + 0.1503	0.99	y = 0.1551x +0.10730	0.99
Average curve		Average curve	
y = 0.004408x + 0.1367	0.99	y = 0.1419x + 0.09598	0.99

**Table 2:** Interval for slope and intercept for each standard curve, with the linearity range. Data were calculated using six different standard curves for each antibody.

Antibodies	Slope		Intercept		Linear Range (ng/mL)
	CI (95%) (ng/mL)	CV	CI (95%) (ng/mL)	CV	
**a-PD-1**	0.00405–0.0047	10.89%	0.1195–0.1539	9.95%	125–2.5
**a-PD-L1**	0.1347–0.1492	12.34%	0.08672–0.105	10.20%	3.125–0.11

CI: confidence interval.

**Table 3:** Statistical analysis of three different mAb concentrations to determine the precision of the (**A**) a-PD-1 ELISA; (**B**) a-PD-L1 ELISA.

**(A)**	**Intra-Day Variability**			**Inter-Day Variability**		
	**62.5 ng/mL**	**15.62 ng/mL**	**3.9 ng/mL**	**62.5 ng/mL**	**15.62 ng/mL**	**3.9 ng/mL**
Average	64.04	15.85	4.06	62.85	16.27	3.82
SD	1.24	2.32	0.52	2.74	2.22	0.53
CV	1.94%	14.66%	12.79%	4.36%	13.64%	13.85%
Imprecision	2.47%	1.51%	4.29%	0.57%	4.19%	−1.90%
**(B)**	**Intra-Day Variability**			**Inter-Day Variability**		
	**3.12 ng/mL**	**0.78 ng/mL**	**0.19 ng/mL**	**3.12 ng/mL**	**0.78 ng/mL**	**0.19 ng/mL**
Average	2.721	0.90	0.19	2.95	0.90	0.21
SD	0.405	0.04	0.02	0.184	0.05	0.03
CV	14.87%	4.87%	9.98%	6.21%	5.36%	16.54%
Imprecision	−12.93%	15.46%	−4.63%	−5.33%	15.84%	7.17%

**Table 4:** Impact on the accuracy and imprecision of the dilution of highest antibody concentrations.

Antibodies	Dilutions			
	1:1	1:2	1:4	1:8
a-PD-1 (62.5 ng/mL)	58.71 ± 0.98	63.83 ± 13.86	64.04 ± 1.86	61.93 ± 4.66
Imprecision	6.06%	2.13%	2.46%	0.91%
a-PD-L1 (3.25 ng/mL)	3.14 ± 0.48	3.25 ± 0.51	3.39 ± 0.18	3.26 ± 1.06
Imprecision	3.38%	0.00%	4.31%	0.31%

**Table 5:** Stability of antibody in plasma samples kept at 4 °C for one month. (ns: non-statistically significant differences).

a-PD-1				a-PD-L1			
ng/mL	Fresh Sample	Day 30	*p*	ng/mL	Fresh Sample	Day 30	*p*
62.5	64.18 ± 8.38	61.81 ± 5.10	ns	3.125	3.53 ± 0.27	3.82 ± 0.44	ns
15.6	17.05 ± 3.65	16.69 ± 2.53	ns	0.781	0.90 ± 0.16	0.81 ± 0.03	ns
3.9	3.258 ± 1.25	3.464 ± 1.75	ns	0.195	0.189 ± 0.01	0.24 ± 0.04	ns

**Table 6:** Drug exposure expressed as AUC_0−∞_ after first dose administration and the parameters, t_1/2_, Cl, and V_d_, calculated by linear regression.

AUC_0−∞_ (µg·h/mL)	t_1/2_(h)	Cl (mL/h)	V_d_ (mL)
2427.35	22.3	0.0823	2.5

**Table 7:** Drug exposure expressed as AUC_0−∞_ after first dose administration and the parameters, t_1/2_, Cl, and V_d_ calculated by linear regression.

AUC_0−∞_ (µg·h/mL)	t_1/2_ (h)	Cl (mL/h)	V_d_ (mL)
1996.42	46.7	0.050	3.3

## Supplementary Materials

The following are available online at https://www.mdpi.com/1999-4923/12/6/595/s1, Figure S1: Antitumor effect of a-PD-1 and a-PD-L1.
