# Supplementary material for: Quantification of Pharmacokinetic Profiles of PD-1/PD-L1 Antibodies by Validated ELISAs
Source: Pharmaceutics. 2020 Jun 26;12(6):595. doi: 10.3390/pharmaceutics12060595 (PMC7356959; doi:10.3390/pharmaceutics12060595)
Supplement: Supplementary file 1 [file pharmaceutics-12-00595-s001.pdf]

pharmaceutics

MDPI

Supplementary Materials: Quantification of Pharmacokinetic Profiles of PD-1/PD-L1 Antibodies by Validated ELISAs

Sara Zalba, Ana M. Contreras-Sandoval, Eva Martisova, Reno Debets, Christian Smerdou and María Jesús Garrido

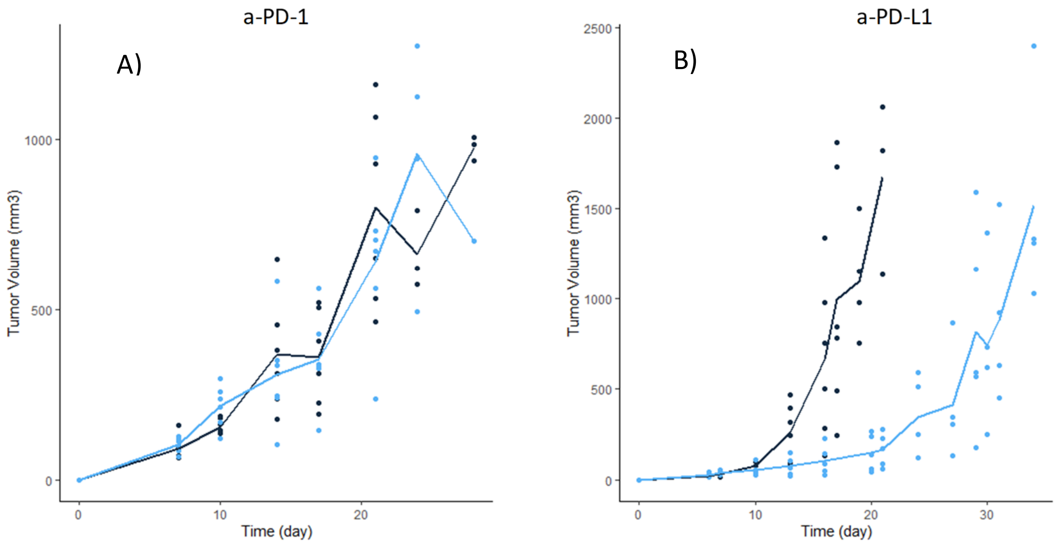

Figure S1. Antitumor effect of a-PD-1 and a-PD-L1 monotherapy in mice. Animals were inoculated with TC-1/A9 (1 × 10^5 cells/100 µL PBS) and B16OVA cells (5 x 10^5 cells/ 100µL PBS) subcutaneously, as is described for the PK study. When tumors achieved 5 mm of diameter, treatments were intravenously administered at days 7, 10, 14 and 17 after tumor inoculation. Tumor growth was measured every two days with an electronic calliper. Tumor volume was calculated with the following formula: Tumor volumen = $(A^2 \times B)/2$, where A represents the shortest diameter and B the longest. Graphs correspond to tumor growth profiles after a-PD-1 and a-PD-L1 treatments, respectively. Control animals are depicted in black and treated animals in blue. (**A**) TC-1 tumor model; (**B**) B16OVA tumor model. In both cases n= 6 per group, although, the number of mice was lower at later time points, in particular in control group, because some mice were sacrificed based on ethical criteria.
